# The bovine dialysable leukocyte extract IMMUNEPOTENT CRP induces immunogenic cell death in breast cancer cells leading to long-term antitumour memory

**DOI:** 10.1038/s41416-020-01256-y

**Published:** 2021-02-03

**Authors:** Alejandra Reyes-Ruiz, Kenny Misael Calvillo-Rodriguez, Ana Carolina Martínez-Torres, Cristina Rodríguez-Padilla

**Affiliations:** 1 Universidad Autónoma de Nuevo León, Facultad de Ciencias Biológicas, Laboratorio de Inmunología y Virología, San Nicolás de los Garza, México; 2 Longeveden, SA de CV, Monterrey, México

**Keywords:** Cancer immunotherapy, Breast cancer

## Abstract

**Background:**

Cancer recurrence is a serious problem in breast cancer (BC) patients, and immunogenic cell death (ICD) has been proposed as a strategy to overcome this recurrence. IMMUNEPOTENT CRP (ICRP) acts as an immunomodulator and can be cytotoxic to cancer cells. Thus, we evaluated if ICRP induces ICD in BC cells.

**Methods:**

Immunogenicity of ICRP-induced cell death was evaluated in vitro, analysing the principal biochemical characteristics of ICD in MCF-7, MDA-MB-231 and 4T1 cells. Ex vivo, we assessed the ability of killed cancer cells (KCC) obtained from ICRP-treated 4T1 cells (ICRP-KCC) to induce DC maturation, T-cell priming and T-cell-mediated cancer cytotoxicity. In vivo, we evaluated tumour establishment and antitumour immune memory after prophylactic ICRP-KCC vaccination in BALB/c mice.

**Results:**

ICRP induced caspase-independent, ROS-dependent cell death, autophagosome formation, P-eIF2α, chaperone protein exposure, CD47 loss, ATP and HMBG1 release in BC cells. Additionally, ICRP-KCC promoted DC maturation, which triggered T-cell priming and cancer cytotoxicity. Prophylactic vaccination with ICRP-KCC prevented tumour establishment and induced long-term antitumour memory in BALB/c mice, involving DC maturation in lymph nodes, CD8+ T-cell augmentation in lymph nodes, peripheral blood and tumour site and ex vivo tumour-specific cytotoxicity by splenocytes.

**Conclusions:**

ICRP induces ICD in BC cells, leading to long-term antitumour memory.

## Background

Breast cancer is the most frequently diagnosed cancer and the leading cause of deaths in women.^1^ One of the principal pitfalls leading to the mortality of this disease is associated with distant metastasis and its ability to recur up to 20 years after diagnosis,^2^ these characteristics are related with the low immunogenicity of breast cancer cells, as a result of cancer cell release of immune-suppressive factors, which block the cancer-immunity cycle.^3^ However, it is now being proposed that with appropriate immune response stimulation, cancer cells could become immunogenic.

In this regard, several studies indicate that immunogenic cell death (ICD) is a hopeful strategy to convert cancer cells into their own vaccine, promising a long-term success of anticancer therapies relying on memory immune response induction,^4,5^ which could deal against high recurrence rate in breast cancer patients. It has been reported that a restricted number of chemotherapies induce ICD,^6^ for instance, breast cancer patients treated with anthracyclines (ICD inducers) showed an increment in the ratio of CD8+ T cells over regulatory T cells intratumourally, and this predicts a favourable therapeutic response.^7^

IMMUNEPOTENT CRP (ICRP), a bovine dialysable leukocyte extract (DLE) obtained from disrupted spleen, is cytotoxic to several cancer cell lines,^8–11^ without affecting the viability of non-cancer cells.^11^ ICRP induces ICD in the murine melanoma model B16F10,^12^ whereas in HeLa and MCF-7 cells, ICRP-mediated cell death involves CRT exposure, ATP and HMGB1 release, which are the principal damage-associated molecular patters (DAMPs) involved in ICD. In addition, ICRP leads to eIF2α phosphorylation (P-eIF2α), which indicates endoplasmic reticulum (ER) stress, an early ICD biomarker. Furthermore, ICRP has been reported to induce reactive oxygen species (ROS)-dependent autophagosome formation in HeLa and MCF-7 cells.^13^

ROS production, ER stress and autophagy stimulate intracellular danger signalling pathways that regulate the release of DAMPs and thus ICD.^14,15^ These results suggest that ICRP might induce ICD in other cancer models, as a conserved mechanism. The aim of this study was to investigate the immunogenicity of ICRP-induced cell death in a panel of breast cancer models, using human and murine cell lines, as well as ex vivo and in vivo experiments using BALB/c mice.

## Methods

### Cell culture

MCF-7 human breast adenocarcinoma (ATCC® HTB-22TM), MDA-MB-231 triple-negative breast adenocarcinoma (ATCC® HTB-26TM) and 4T1 murine mammary adenocarcinoma (ATCC® CRL2539TM) cell lines were obtained from the American Type Culture Collection. MCF-7 and MDA-MB-231 cells were cultured in DMEM-F12 supplemented with 10% foetal bovine serum (FBS) and 1% penicillin–streptomycin (complete DMEM), and 4T1 cells were cultured in RPMI-1640 supplemented with 10% FBS and 1% penicillin–streptomycin (complete RPMI) (Life Technologies, Grand Island, NY) and routinely grown in plastic tissue-culture dishes (Life Sciences, Corning, NY). All cell cultures were maintained in a humidified incubator in 5% CO_2_ at 37 °C. Cell count was performed in a Neubauer chamber, using 0.4% trypan blue (MERCK, Darmstadt, Germany).

### Animals

The Animal Research and Welfare Ethics Committee (CEIBA), of the School of Biological Sciences approved this study: CEIBA-2019-006. All experiments were conducted according to Mexican regulation NOM-062-ZOO-1999. Female BALB/c mice (six-to-eight-week-old, 20 ± 2 g of weight) were provided by the animal house at the Universidad Autonoma de Nuevo Leon, Mexico. Animals were housed in plastic cages in groups of four and given seven days to acclimate to the housing facility. Environmental conditions were temperature 21 °C ± 3 °C, humidity 55% ± 10% and 12-h light/dark cycle. Animals were supplied with rodent maintenance food (LabDiet, St. Louis, MO) and water ad libitum, and they were monitored twice daily for health status, no adverse events were observed. Mice were randomly assigned to different groups for all studies. All experiments were designed in accordance to the Arrive guidelines for animal care and protection (Methods S1).^16^

### Cell death assay

Cell death was measured using 1 µg/mL APC Annexin V (BD Pharmingen, San Jose, CA) and 0.5 µg/mL propidium iodide (PI) (MERCK). In brief, 5 × 10^4^ cells/well in 24-well dishes (Life Sciences) were incubated in the presence or absence of different concentrations of IMMUNEPOTENT CRP (ICRP), epirubicin (EPI, Sigma-Aldrich, ST. Louis, MO) or cyclophosphamide (CPA, Sigma-Aldrich) for 24 h in complete DMEM or RPMI to a final volume of 400 μL to obtain the ICRP CC_50_. For cell death inhibition assays, cells were pre-treated 30 min with or without 5mM N-acetyl-L-cysteine (NAC), 10 μM QVD.oph, 30 μM Necrostatin-1 (NEC-1) or 15 μM Spautin-1 (Sp-1), and then co-treated with or without ICRP CC_50_ for 24 h, as mentioned before. Cells were then detached, washed twice with PBS, suspended in 100 μL of binding buffer (10 mM HEPES/NaOH, pH 7.4, 140 mM NaCl and 2.5 mM CaCl_2_), stained and assessed by BD Accury C6 flow cytometer (Becton Dickinson, Franklin Lakes, NJ). The results were analysed using FlowJo Software (LLC, Ashland, OR).

### Loss of mitochondrial membrane potential analysis

For these assays, cells were stained with 500 nM TMRE (Sigma-Aldrich) to evaluate the loss of mitochondrial membrane potential. In brief, 5 × 10^4^ cells/well in 24-well dishes (Life Sciences) were incubated in the presence or absence of ICRP CC_50_ for 24 h, as explained above. Cells were then detached, washed with PBS, stained, incubated at 37 °C for 30 min and measured by flow cytometry as described above.

### ROS generation analysis

ROS levels were determined by staining cells with 2.5 μM 2′,7′-dichlorofluorescin diacetate (DCFDA) (MERCK). In brief, 5 × 10^4^ cells/well were incubated in the presence or absence of ICRP CC_50_ for 24 h, as explained above. Cells were then detached, washed with PBS, stained, incubated at 37 °C for 30 min and measured using a flow cytometer, as mentioned above.

### Autophagosome formation assays

For flow cytometric assessment, 5 × 10^4^ cells were cultured in 24-well plates (Life Sciences) in the presence or absence of ICRP CC_50_ for 24 h. Cells were then detached, washed with PBS, stained with Autophagy Detection Kit (Abcam, Cambridge, UK) and measured by flow cytometry, as explained above.

For fluorescence microscopy, 5 × 10^4^ cells were cultured in 24-well plates (Life Sciences), pre-treated 30 min with or without 15 μM Sp-1 and then co-treated with or without ICRP CC_50_ for 24 h, as mentioned above. Cells were then washed twice with PBS and stained with CYTO-ID Autophagy Detection Kit (Enzo Life Science, Farmingdale, NY) following the manufacturer’s instructions. Nuclei were counterstained in blue with Hoechst 33342 dye (Enzo Life Science). Images were acquired with an InCellis Imager (BERTIN Instruments, Montigny-le-Bretonneux, France), fluorescence was measured using Image J (NIH Image, Bethesda, MD) to obtain the corrected total cell fluorescence (CTCF) using the following formula: CTCF = integrated density − (area of selected cell * mean fluorescence of background readings).

### Western blot analysis

Cells were treated with ICRP CC_50_ for 24 h and were lysed in lysis buffer containing 20 mM Tris (pH 6.8), 2% SDS, 2 mM EDTA and 300 mM NaCl. The protein concentration for each sample was determined using a spectrophotometer in accordance with the manufacturer’s recommendations. The proteins were separated by SDS-PAGE and transferred to nitrocellulose membranes. Western blotting was performed with anti-p62 (sc-48402, Santa Cruz Biotechnology, Dallas, TX) and anti-β-actin (BA3R, abm, Richmond, BC). After incubation with the anti-mouse secondary antibodies conjugated to horseradish peroxidase (ab6728, Abcam), blots were revealed using ECL western blotting substrate (Thermo Scientific, Waltham, MA).

### EIF2α assay

For this assay, 5 × 10^5^ cells were plated in 6-well dishes (Life Sciences) in complete DMEM or RPMI to a final volume of 2 mL, and incubated in the presence or absence of ICRP CC_50_ for 18 h or 1 μM thapsigargin (thaps) for 2 h. Cells were then collected and fixed with 80% methanol for 5 min at −20 °C, washed with PBS and permeabilized with 0.1% PBS-Tween for 20 min at 25 °C. The cells were then washed with PBS, suspended in 50 μL of 1× PBS/10% FSB/0.3 M glycine, incubated for 30 min and shaken at 400 rpm, 25 °C, followed by the addition of 0.2 μL of anti-EIF2S1 (phospho S51) antibody [E90] (ab32157, Abcam) (1:250) for eIF2α phosphorylation (P-eIF2α) analysis or 0.5 μL of anti-EIF2A (3A7A8, Santa Cruz Biotechnology) (1:100) for eIF2α total (T-eIF2α) assays, cells were incubated for 2 h and washed with 2% FACS Buffer (1× PBS and 2% FBS). Cells were then suspended in 100 μL of 1× PBS/10% FSB/0.3 M glycine, incubated for 15 min and shaken at 400 rpm and 25 °C; 0.2 μL of goat anti-rabbit IgG H&L (Alexa Fluor® 488) (ab150077, Abcam) (1:500) or 0.2 μL of goat anti-mouse IgG (Alexa Fluor® 488) (H+L, Life Technologies) (1:500) were then added for P-eIF2α or T-eIF2α analyses respectively, and incubated for 1 h in darkness. Cells were washed with 2% FACS Buffer and the fluorescence was measured by flow cytometry, as mentioned before.

### Calreticulin, HSP70, HSP90 and CD47 exposure analyses

For this, 5 × 10^4^ cells were seeded in 24-well plates and treated with ICRP CC_50_, EPI CC_50_ or CPA CC_50_ for 24 h. Cells were detached, washed and incubated for 1 h at 25 °C with 2 μg/mL Calreticulin-antibody (FMC-75, Enzo Life Science), 0.8 μg/mL HSP70-antibody (F-3, Santa Cruz Biotechnology), 0.8 μg/mL HSP90-antibody (F-8, Santa Cruz Biotechnology), 3 μg/mL CD47 human-antibody (B6H12, Santa Cruz Biotechnology) or 3 μg/mL CD47 mouse-antibody (MIAP301, Santa Cruz Biotechnology), in 2% FACS buffer, cells were washed and incubated for 30 min in darkness at 25 °C with goat anti-mouse IgG (Alexa Fluor 488) (H + L, Life Technologies) (1:1500) or anti-rat IgG (Alexa Fluor 488) (H + L, Life Technologies) (1:1500) in 2% FACS buffer, cells were then washed, suspended in 2% FACS buffer with 7-AAD (Life Technologies) (1:1000) or Fixable viability stain 780 (BD Biosciences) (1:1000) and incubated protected from light for 10 min at 25 °C. The surface exposure of CRT, HSP70, HSP90 and CD47 was determined by flow cytometry among viable (7-AAD-negative or Fixable viability stain 780-negative) cells.

### ATP release assay

Supernatants of ICRP, EPI or CPA CC_50_-treated cells (2 × 10^5^) were used to assess extracellular ATP by a luciferase assay (ENLITEN kit, Promega, Madison, WI), following the manufacturer’s instructions. Bioluminescence was determined in a Synergy HT microplate reader, using the Software Gen5 (BioTek, Winooski, VT) at 560 nm.

### High-mobility group box 1 release assay

For this assay, 2 × 10^5^ cells were treated with ICRP or EPI CC_50_ for 24 h or 48 h. Supernatants were used to assess extracellular HMGB1 using the HMGB1 BioAssay ELISA Kit (Human) for MCF-7 and MDA-MB-231 cells (US Biological Life Science, Salem, MA), and the HMGB1 BioAssay ELISA Kit (Mouse) for 4T1 cells (US Biological Life Science), following the manufacturer’s instructions, in a spectrophotometer at a wavelength of 450 nm.

### Generation of mouse BMDCs

To obtain bone marrow-derived dendritic cells (BMDCs), seven-to-eight-week-old BALB/c mice were anaesthetised with an intraperitoneal injection of ketamine (80 mg/kg body weight) and xylazine (10 mg/kg body weight) and were euthanised by cervical dislocation (*n* = 5 mice). Bone marrow was removed from femur and tibia after mouse death by flushing into complete RPMI (Life Technologies), as previously described.^17^ Eluted cells were cultured at 37 °C in a controlled humidified atmosphere with 5% CO_2_ for 5 days in complete RPMI and 20 ng/mL IL-4 and GM-CSF (R&D Systems, Minneapolis, MN), until approximately 50% of cells were CD11c+.

### T-cell isolation

Seven-to-eight-week-old BALB/c mice were anaesthetised as mentioned above. Blood was obtained by cardiac puncture, and then cervical dislocation was performed. Peripheral blood mononuclear cell (PBMC) isolation was performed by density-gradient centrifugation, using Ficoll-Hypaque-1119 (MERCK). CD3+ cells were isolated from total PBMCs by positive selection using magnetic-activated cell sorting microbead technology with anti-CD3+-biotin and anti-biotin microbeads (Miltenyi Biotec, Bergisch Gladbach, Germany), following the manufacturer’s instructions. Primary murine CD3+ cells were maintained in complete RPMI and incubated at 37 °C in a controlled humidified atmosphere with 5% CO_2_.

### Killed cancer cell (KCC) obtention

For the obtention of 4T1 killed cancer cells (KCC), we treated 4T1 cells with CC_100_ of ICRP (0.5 U/mL, ICRP-KCC), CC_100_ of EPI (10 μM, EPI-KCC) or CC_100_ of CPA (35 mM, CPA-KCC), and the dead cells obtained were then washed and detached; cell death (>90%) was confirmed using trypan blue staining and flow cytometry.

### ICRP-KCC-mediated BMDC maturation

BMDCs were suspended in complete RPMI at a concentration of 1 × 10^6^ cells/mL and stimulated with ICRP-killed cancer cells (ICRP-KCC) (3 × 10^6^ cells/mL at a ratio of 1:3 BMDCs to ICRP-treated 4T1 cells (BMDC-ICRP-KCC)). Control BMDCs were left untreated or stimulated with 1 μg/mL lipopolysaccharide (LPS) (MERCK). After 24 h, culture supernatants were removed and stored at –80 °C to analyse TNF-α release by flow cytometry (BD CBA Mouse Th1/Th2 Cytokine Kit, BD Biosciences, San Jose, CA), and wells were washed twice with PBS before the next co-culture (with the addition of T lymphocytes). Additionally, some BMDC-Control and BMDC-ICRP-KCC wells were collected to analyse DC marker expression.

### DC marker expression

For this assay, 1 × 10^5^ BMDCs were suspended in 100 μL of 2% FACS buffer, and maturation was analysed by immunostaining using anti-CD11c-Alexa Fluor 488 (R&D Systems), anti-CD80-FITC and anti-CD86-APC (BD Biosciences) at 25 °C for 30 min, and washed twice with PBS. Cell surface markers were evaluated by flow cytometry, as mentioned above.

### BMDC T-lymphocyte co-culture

BMDC-Control or BMDC-ICRP-KCC were maintained in complete RPMI at a concentration of 1 × 10^6^ cells/mL. Allogeneic BALB/c mCD3+ cells were then added to each well at 3 × 10^6^ cells/mL (at a ratio of 1:3 DCs to CD3+ cells), and incubated for 96 h at 5% CO_2_ and 37 °C. Supernatants were then removed, stored at −80 °C to further analyse TNF-α, IFN-γ, IL-4 and IL-5 levels by flow cytometry (BD CBA Mouse Th1/Th2 Cytokine Kit, BD Biosciences), and lymphocytes were washed with PBS, and suspended in complete RPMI to be used in the next co-culture (T lymphocytes with cancer cells).

### T lymphocyte–4T1 cell co-culture

Viable 4T1 cells were seeded at a concentration of 1 × 10^5^ cells/mL. Cells were then stained with 0.1 μL/mL of calcein-AM (BD Biosciences) for 30 min and washed twice with PBS. Next, unprimed (previously co-cultured with BMDC-Control) or primed (previously co-cultured with BMDC-ICRP-KCC) allogeneic BALB/c CD3+ cells were added to each well at 5 × 10^5^ cells/mL (at a ratio of 1:5 cancer cells to CD3+ cells); 4T1–T-lymphocyte co-culture was incubated at 37 °C and 5% CO_2_ for 24 h. Supernatants were removed and stored at −80°C to further analyse IFN-γ, IL-4 and IL-5 levels, as mentioned above. Cancer cells were then washed and detached to analyse 4T1-calcein-negative cells by flow cytometry, as described above.

### Prophylactic vaccination

For this, 4T1 cells were treated with 0.5 U/mL ICRP, 10 μM EPI or 35 mM CPA for 24 h; cells were then washed, detached and cell death was confirmed using trypan blue staining and flow cytometry, as previously indicated to obtain the KCC. Seven-to-eight-week-old BALB/c mice were inoculated subcutaneously (s.c.) with 1.5 × 10^6^ ICRP-killed 4T1 cells (ICRP-KCC, *n* = 10 mice), 1.5 × 10^6^ EPI-killed 4T1 cells (EPI-KCC, *n* = 10 mice), 1.5 × 10^6^ CPA-killed 4T1 cells (CPA-KCC, *n* = 10 mice) or with PBS (*n* = 10 mice) on the left flank side. On day 7 after vaccination, the mice were challenged s.c. on the opposite flank with 5 × 10^5^ viable 4T1 cells.

### Tumour volume measurements

Tumour volume was measured three times a week, using a calliper (Digimatic Calliper Mitutoyo Corporation, Japan), this was determined with the following formula: tumour volume (mm^3^) = 4+/3 ∗ A (length) ∗ B (width) ∗ C (height). When the tumour reached the parameters that confine mouse pain and distress, or mice presented two or more parameters of pain and distress, as postulated by the Institutional Animal Research and Welfare Ethics Committee (CEIBA), mice were anaesthetised as described above and were euthanised by cervical dislocation.

### Long-term memory assays

Mice in complete remission after ICRP-KCC prophylactic vaccination were rechallenged with 5 × 10^5^ viable 4T1 cells in 100 μL of PBS into the opposite flank (*n* = 9 mice), and naive mice were used as control (*n* = 9 mice). Tumour volume and mice survival were evaluated, as described above.

Additionally, 3 days after tumour rechallenge, tissues from tumour-draining lymph nodes (TDLN) and tumour re-challenge sites were obtained from naive and ICRP-KCC mice, and fixed in 3.7% neutral formalin, embedded in paraffin, sectioned (5-μm thickness) and stained with H&E (MERCK). Histopathological analyses were done by an external veterinarian pathologist (National professional certificate 2593012). Blood was obtained by cardiac puncture from anaesthetised mice as described above, and PBMC isolation was performed by density-gradient centrifugation, using Ficoll-Hypaque-1119 (MERCK). Cells from tumour rechallenge site, TDLN and spleen were isolated using 70-μm cell strainers (MERCK) and suspended in 2% FACS buffer. PBMCs, TDLN cells and tumour rechallenge site cells were stained with Mouse T lymphocyte antibody cocktail: PE-Cy7 CD3e, PE CD4 and FITC CD8 (BD Pharmingen) following the manufacturer’s instructions. Maturation of DCs was analysed by immunostaining of cells from lymph nodes using anti-CD11c-Alexafluor 488 (R&D Systems), and anti-CD86-APC (BD Biosciences) at 25 °C for 30 min and washed twice with PBS. Cell surface markers were evaluated by flow cytometry, as mentioned above. Viable 4T1 cells were seeded at a concentration of 1 × 10^5^ cells/mL, stained with 0.1 μL/mL of calcein-AM (BD Biosciences) for 30 min and washed twice with PBS. Next, splenocytes from naive or ICRP-KCC mice were added to each well at 40 × 10^5^ cells/mL (at a ratio of 1:40 cancer cells to splenocytes); 4T1-splenocyte co-culture was incubated at 37 °C and 5% CO_2_ for 24 h. Supernatants were removed and stored at −80 °C to further analyse IFN-γ, TNF-α, IL-2, IL-4 and IL-5 levels, as mentioned above. Cancer cells were then washed and detached to analyse 4T1-calcein-negative cells by flow cytometry, as described above (*n* = 6 mice per group).

### Statistical analysis

Data were analysed using GraphPad Prism Software (GraphPad Software Inc., San Diego, CA) and shown as mean ± SD of triplicates from three independent experiments. For in vitro studies, statistical analyses were done using paired Student’s *t*-test, and for in vivo and ex vivo studies, Mann–Whitney tests and two-tailed unpaired Student’s *t*-tests were performed.

## Results

### IMMUNEPOTENT CRP induces loss of mitochondrial membrane potential, pro-survival autophagosome formation and ROS-dependent cell death in MCF-7, MDA-MB-231 and 4T1 cells

**Fig. 1:**
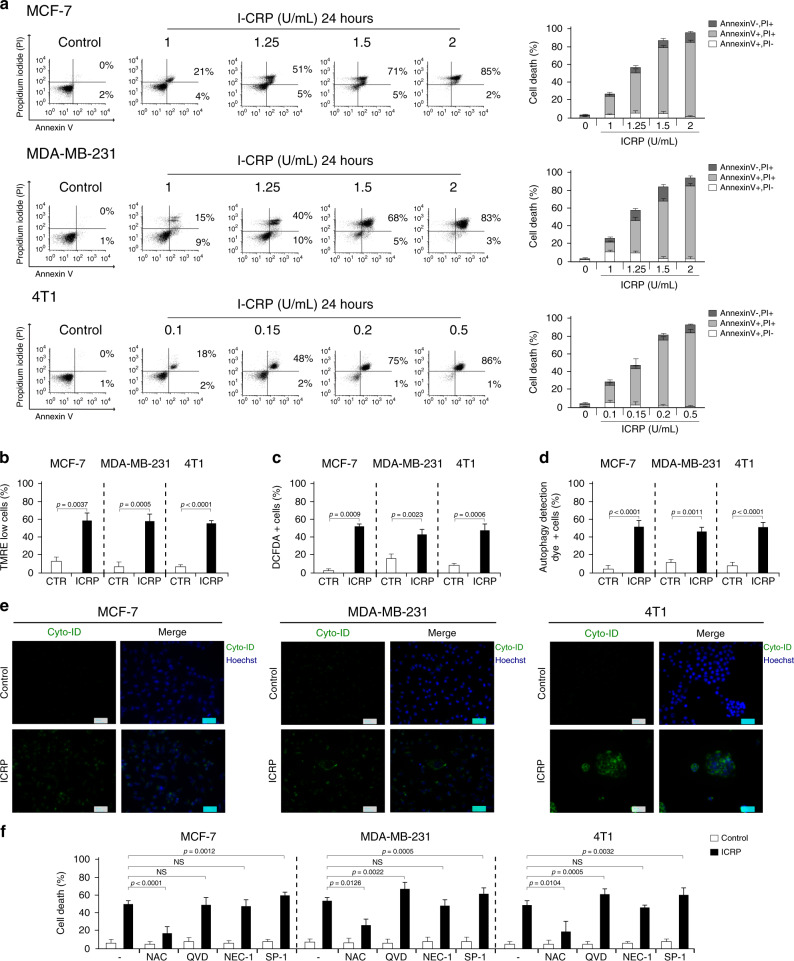
IMMUNEPOTENT CRP induces loss of mitochondrial membrane potential, pro-survival autophagosome formation and ROS-dependent cell death in MCF-7, MDA-MB-231 and 4T1 cells. **a** Representative dot plots (left) and quantifications (right) of cell death measured by flow cytometry through Annexin-V and PI staining in MCF-7, MDA-MB-231 and 4T1 cells treated with different concentrations of ICRP for 24 h. **b**–**d** Quantifications of loss of mitochondrial membrane potential evaluated through TMRE staining (**b**) ROS levels assessed through DCFDA staining (**c**) or autophagosome formation measured through Green Detection Reagent staining (**d**) by flow cytometry in the absence (control, shown in white bar) or presence (shown in black bar) of ICRP CC_50_ for 24 h in MCF-7, MDA-MB-231 and 4T1 cells. **e** Representative immunofluorescence images of CYTO-ID staining in MCF-7, MDA-MB-231 and 4T1 cells left untreated (control) or treated with ICRP CC_50_ for 24 h. Nuclei were counterstained in blue with Hoechst 33342 dye. **f** Quantification of cell death (Annexin V−, PI+, Annexin V+, PI+, Annexin V+ and PI−) measured by flow cytometry through Annexin-V and PI staining in MCF-7, MDA-MB-231 and 4T1 cells in the absence (control, shown in white bar) or presence (shown in black bar) of ICRP CC_50_ for 24 h without co-treatment (−) or co-treated with N-acetyl cystein (NAC), QVD-OPH (QVD), Necrostatin-1 (NEC-1) or Spautin-1 (SP-1). The means (±SD) of triplicates of at least three independent experiments were graphed.

ICRP induced cell death in all cell lines, in a concentration-dependent manner, as shown in Fig. 1a. Cell death in 30% of the cells (CC_30_) was reached at 1 U/mL in MCF-7 and MDA-MB-231, and 0.1 U/mL in 4T1 cells. CC_50_ by ICRP was caused at 1.25 U/mL in MCF-7 and MDA-MB-231 cells, and 0.15 U/mL in 4T1 cells, whereas CC_80_ was induced at 1.5 U/mL in human breast cancer cells, and 0.2 U/mL in 4T1 cells, CC_100_ was reached at 2 U/mL in MCF-7 and MDA-MB-231, and 0.5 U/mL in 4T1 cells, where we observed more than 90% of cell death (Fig. 1a). The ICRP-killed 4T1 cells (ICRP-KCC) were generated with this CC_100_. Loss of mitochondrial membrane potential was induced in 55–65% of MCF-7, MDA-MB-231 and 4T1 cells treated with ICRP CC_50_ for 24 h (Fig. 1b), also, this treatment generated an increase of ROS levels in 45–55% of MCF-7, MDA-MB-231 and 4T1 cells (Fig. 1c). Moreover, we observed autophagosome formation in 40–55% of MCF-7, MDA-MB-231 and 4T1 cells assessed by flow cytometry (Fig. 1d). This autophagosome formation was confirmed by fluorescence microscopy (Fig. 1e), which was inhibited in the presence of the autophagy inhibitor Sp-1 (Supplementary Fig. 1A). In addition, p62 downregulation was induced in MCF-7, MDA-MB-231 and 4T1 cells treated with ICRP CC_50_ for 24 h (Supplementary Fig. 1B).

The ROS scavenger NAC significantly inhibited ICRP-induced cell death, whereas the autophagy inhibitor Sp-1 significantly potentiated ICRP-mediated cell death (Fig. 1f). No significant changes were observed with the necroptosis inhibitor NEC-1 in MCF-7, MDA-MB-231 and 4T1 cells (Fig. 1f). Additionally, the caspase inhibitor QVD significantly potentiated ICRP-mediated cell death in MDA-MB-231 and 4T1 cells, whereas no significant changes were observed in MCF-7 cells (Fig. 1f).

### IMMUNEPOTENT CRP causes eIF2α phosphorylation and DAMP emission in MCF-7, MDA-MB-231 and 4T1 cells

**Fig. 2:**
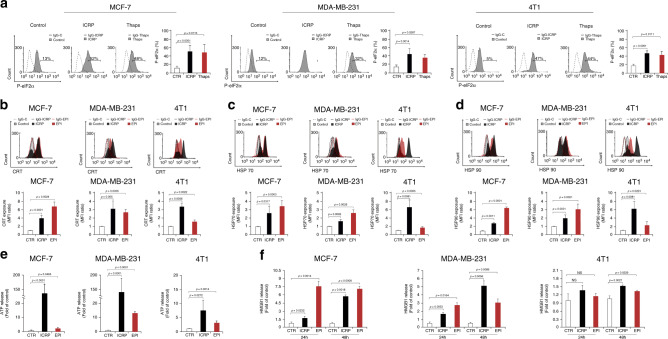
IMMUNEPOTENT CRP causes eIF2α phosphorylation and DAMP emission in MCF-7, MDA-MB-231 and 4T1 cells. **a** Representative FACS histograms of P-eIF2α staining (in grey) and IgG isotype antibodies (dotted) of cancer cells left untreated (negative control) or treated with ICRP CC_50_ for 18 h or 1 μM Thaps for 2 h (positive control). Charts are the quantification of P-eIF2α staining in controls and cancer cells treated with ICRP or Thaps. **b**–**d** Representative FACS histograms of CRT (**b**), HSP70 (**c**) or HSP90 (**d**) exposure (filled histograms) and IgG isotype antibodies (open histograms) of cancer cells left untreated (negative control in grey) or treated with ICRP CC_50_ (in black), or EPI CC_50_ (positive control in red) for 24 h. Charts are the quantification of CRT (**b**), HSP70 (**c**) or HSP90 (**d**) exposure in cancer cells left untreated or treated with ICRP CC_50_ or EPI CC_50_ for 24 h. **e** Quantification of ATP release through bioluminescence detection in the supernatants of cancer cells in the absence (negative control) or presence of ICRP CC_50_ or EPI CC_50_ for 24 h. **f** Quantification of HMGB1 release assessed by ELISA in the supernatants of cancer cells in the absence (negative control) or presence of ICRP CC_50_ or EPI CC_50_ for 24 h and 48 h. Graphs represent the means (±SD) of triplicates of at least three independent experiments.

ICRP induced P-eIF2α in 40–60% of all cell lines treated with ICRP CC_50_ for 18 h; similar results were found when cells were treated with 1 μM Thaps for 2 h (Fig. 2a); importantly, no changes in total eIF2α expression (T-eIF2α) were found after treatments (Supplementary Fig. 2).

In addition, ICRP CC_50_ treatment for 24 h induced 3.73 ± 0.83-, 3.07 ± 0.97- and 3.20 ± 0.59-fold CRT exposure (Fig. 2b), 2.61 ± 0.82-, 1.63 ± 0.24- and 6.51 ± 1.74-fold HSP70 exposure (Fig. 2c), 2.55 ± 0.26-, 1.93 ± 0.31- and 6.23 ± 1.35-fold HSP90 exposure (Fig. 2d) and 135 ± 31.38-, 140.99 ± 50.38- and 7.30 ± 3.92-fold ATP release (Fig. 2e) in MCF-7, MDA-MB-231 and 4T1 cells, respectively, when compared with untreated cells. ICRP-treated cells also presented a decrease in CD47 exposure of 0.66 ± 0.21-, 0.79 ± 0.07- and 0.49 ± 0.17-fold in MCF-7, MDA-MB-231 and 4T1 cells, respectively, as compared with untreated control (Supplementary Fig. 3). Additionally, EPI CC_50_ treatment for 24 h induced 6.61 ± 1.61-, 2.69 ± 0.33- and 1.46 ± 0.20-fold CRT exposure (Fig. 2b), 3.41 ± 0.64-, 2.56 ± 0.45- and 1.66 ± 0.19-fold HSP70 exposure (Fig. 2c), 6.24 ± 0.28-, 2.98 ± 0.69- and 2.29 ± 0.95-fold HSP90 exposure (Fig. 2d) and 2.02 ± 0.92-, 12.61 ± 1.47- and 3.07 ± 0.66-fold ATP release (Fig. 2e) in MCF-7, MDA-MB-231 and 4T1 cells, respectively, in contrast with untreated cells.

Our results also revealed that ICRP CC_50_ treatment for 24 h induced 1.58 ± 0.07-, 1.65 ± 0.25- and 1.34 ± 0.28-fold HMGB1 release, and the same treatment for 48 h triggered 5.32 ± 0.83-, 4.94 ± 1.14- and 1.49 ± 0.04-fold HMGB1 release in MCF-7, MDA-MB-231 and 4T1 cells, respectively, as compared with untreated cells. Finally, EPI CC_50_ treatment for 24 h caused 7.51 ± 1.12-, 2.66 ± 0.61- and 1.13 ± 0.11-fold HMGB1 release, and this treatment for 48 h provoked 7.55 ± 0.89-, 2.87 ± 0.61- and 1.29 ± 0.08-fold HMGB1 release in MCF-7, MDA-MB-231 and 4T1 cells, respectively, in contrast with untreated cells (Fig. 2f).

Interestingly, CPA CC_50_ treatment for 24 h only induced significative DAMP emission in MCF-7 cells, but not MDA-MB-231 and 4T1 cells (Supplementary. Fig. 4)

### Dead tumour cells obtained after ICRP treatment induce maturation of BMDCs

**Fig. 3:**
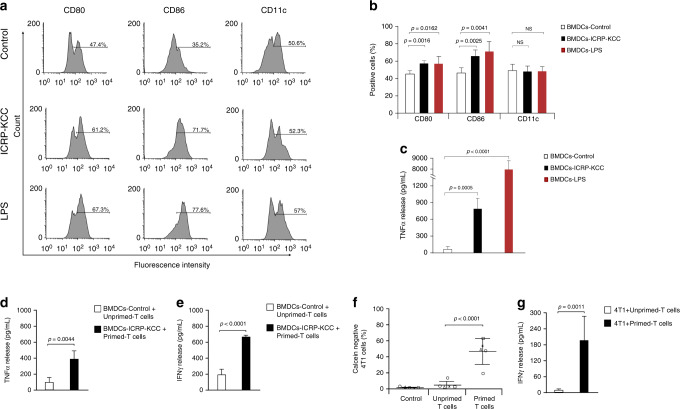
ICRP-KCC triggers BMDC maturation and anticancer immune response. **a** Representative flow cytometry histograms showing the percentage of CD80+, CD86+ and CD11c+ BMDCs unstimulated (negative control), in co-culture ratio 1:3 with ICRP-treated 4T1 cells (ICRP-KCC) or stimulated with 1 μg/mL of LPS (positive control) during 24 h. **b** BMDCs were treated as in (**a**) and the means (±SD) obtained of five independent experiments were graphed. **c** Quantification of TNF-α concentration in supernatants of BMDCs treated as in (**a**). **d**, **e** Concentration of TNF-α (**d**) and IFN-γ (**e**) in supernatants of unstimulated BMDCs (BMDC-Control) or stimulated with ICRP-KCC (BMDC-ICRP-KCC), in co-culture ratio 1:3 with T cells for 96 h, expressed as the means (±SD) of five independent experiments. **f** Percentage of calcein-negative 4T1 cells left alone (Control) or after co-culture with unprimed T lymphocytes (previously co-cultured with BMDC-Control) or primed T lymphocytes (previously co-cultured with BMDC-ICRP-KCC) for 24 h (co-culture ratio 1:5). **g** Quantification of IFN-γ concentration in supernatants of 4T1 cells treated as in (**f**), expressed as the means (±SD) of five independent experiments. *n* = 5 mice per group.

After determining that the principal biochemical characteristics of immunogenic cell death were evoked after ICRP treatment, we evaluated the capacity of the dead cells (obtained after ICRP (CC_100_) treatment, ICRP-KCC) to mature BMDCs. As observed in Fig. 3a, b, the exposure of BMDCs to ICRP-KCC (BMDC-ICRP-KCC) significantly increased the expression of CD80 (57%), and CD86 (65%), whereas it maintained CD11c expression (48%), in comparison with unstimulated BMDCs (C80: 44%, CD86: 45% and CD11c: 49%). These results resemble the ones observed by our positive control, LPS, which significantly incremented CD80 (56%) and CD86 expression (70%), and also maintained CD11c expression (48%) in these cells. Furthermore, a significant increase in TNF-α release was observed in BMDCs stimulated with ICRP-KCC (760.68 ± 209.84 pg/mL) or LPS (7763.41 ± 1158.50 pg/mL), in comparison with unstimulated BMDCs (52.72 ± 1.66 pg/mL) (Fig. 3c). Thus, ICRP-KCC induced BMDC maturation.

### Mature BMDCs exposed to ICRP-tumour cell lysate trigger anticancer immune responses

After the evaluation of ICRP-KCC-mediated BMDC maturation, the next step was to investigate if these mature cells could induce T-cell priming. Figure 3d, e shows a significant increase of TNF-α (380 ± 139.48 pg/mL) and IFN-γ (650.37 ± 12.86 pg/mL) release in the co-culture of BMDC-ICRP-KCC and T-cells, in contrast to the co-culture of BMDC-Control and T cells (TNF-α: 95.89 ± 71.63 pg/mL and IFN-γ: 185.67 ± 75.09 pg/mL). No significant differences were detected in TH-2 cytokines, IL-4 and IL-5 release (Supplementary. Table 1). Primed T cells obtained after co-culture with BMDC-ICRP-KCC caused a cytotoxic effect in up to 70% of 4T1 cells, whereas the cytotoxicity induced by unprimed T cells was up to 19% of cancer cells; no cytotoxicity was detected in 4T1 cells without T cells (Fig. 3f and Supplementary. Fig. 5). In addition, a significant increase in IFN-γ release was observed in the co-culture of primed T cells with 4T1 cancer cells (192.29 ± 96.54 pg/mL), in comparison with the co-culture of unprimed T cells with 4T1 cells (8.44 ± 4.55 pg/mL) (Fig. 3g). No significant differences were detected in TH-2 cytokines, IL-4 and IL-5 release (Supplementary Table 1). These data confirm the efficient antigen presentation by BMDC-ICRP-KCC to T cells and the immunocompetence of these T cells against 4T1 cells.

### Prophylactic vaccination with ICRP-KCC prevents tumour establishment in BALB/c mice

**Fig. 4:**
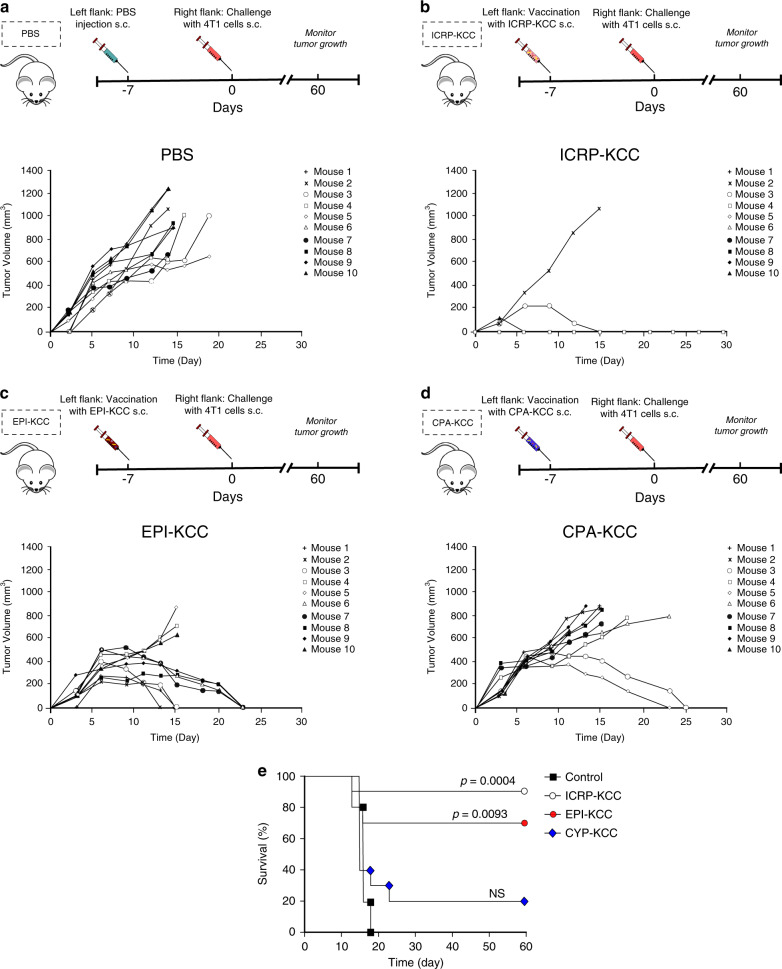
Prophylactic vaccination with ICRP-KCC prevents tumour establishment in BALB/c mice. **a**–**d** Mice were inoculated s.c. with PBS, 1.5 × 10^6^ ICRP-killed 4T1 cells (ICRP-KCC), 1.5 × 10^6^ EPI-killed 4T1 cells (EPI-KCC) or 1.5 × 10^6^ CPA-killed 4T1 cells (CPA-KCC) on the left flank side. On day 7 after vaccination, mice were challenged s.c. on the opposite flank with 5 × 10^5^ viable 4T1 cells. Tumour growth on the challenge site was evaluated for up to 60 days after the challenge. Tumour volume on the challenge site of unvaccinated mice (PBS) (**a**) or vaccinated with ICRP-KCC (**b**), EPI-KCC (**c**) or CPA-KCC (**d**). Each line represents one mouse. **e** Kaplan–Meier graph with the percentage of survival in mice treated as in (**a**–**d**) (*n* = 10 mice per group).

In order to test the ability of ICRP-KCC to activate adaptive immune system in vivo, we performed a well-established prophylactic tumour vaccination model in immunocompetent BALB/c mice^4,18^ (Fig. 4). All unvaccinated (PBS) mice reached a correct tumour establishment on the challenge site (Fig. 4a), while immunisation of mice with ICRP-KCC prevented tumour growth at the challenge site in nine out of ten mice (Fig. 4b). Additionally, vaccinated mice with EPI-KCC induced tumour regression in 7/10 mice (Fig. 4c), while vaccination with CPA-KCC induced tumour regression at the challenge site in only 2/10 mice (Fig. 4d). These data confirmed that ICRP-KCC induced a potent immune response in vivo, reflected in 90% (9/10) of 60-day survival rates of mice in the ICRP-KCC group, whereas EPI induced 70% (7/10) 60-day survival, CPA induced 20% (2/10) survival and all PBS mice were euthanised by day 20 (10/10) (Fig. 4e).

### Prophylactic vaccination with ICRP-KCC induces long-term antitumour memory in BALB/c mice

**Fig. 5:**
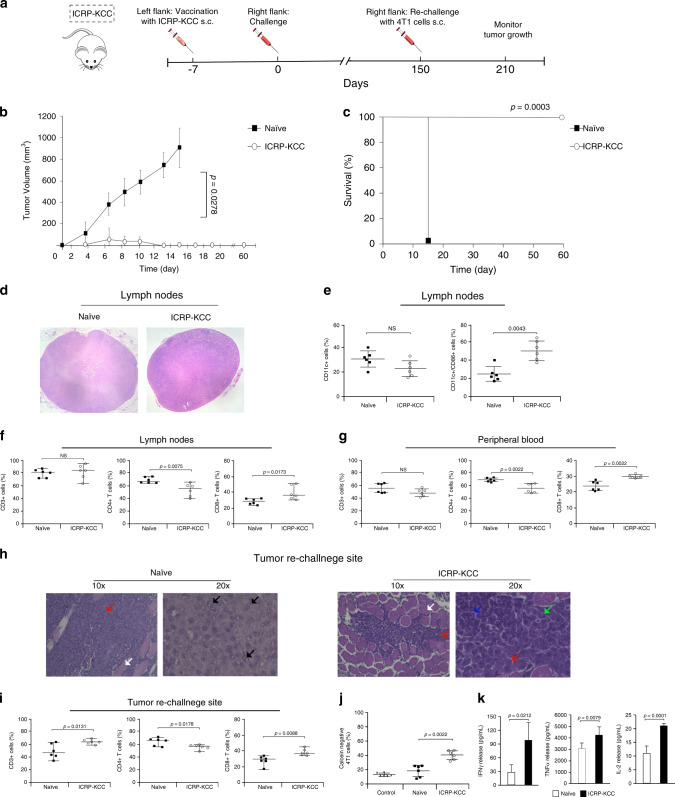
ICRP-KCC prophylactic vaccination induces long-term antitumour memory, modulates tumour establishment, DC maturation, T-cell distribution and splenocyte–tumour-specific cytotoxicity after tumour rechallenge. **a** Mice in remission after ICRP-KCC prophylactic vaccination were rechallenged s.c. with 5 × 10^5^ viable 4T1 cells after 150 days of prophylactic vaccination. Tumour growth on the challenge site was evaluated for up to 60 days after rechallenge. **b** Tumour volume on the challenge site of naive mice (black square, *n* = 9 mice) or mice in remission after a previous 1.5 × 10^6^ ICRP-KCC vaccination (white circle, *n* = 9 mice). **c** Kaplan–Meier graph with the percentage of survival of mice treated as in (**c**). **d**, **k** Naive mice (*n* = 6) and mice in remission after ICRP-KCC prophylactic vaccination (*n* = 6) were challenged/rechallenged s.c. with 5 × 10^5^ viable 4T1 cells. Three days later, tumour-draining lymph nodes, peripheral blood, the tumour rechallenge site and spleen were obtained. **d** Histology from lymph nodes of naive and ICRP-KCC mice stained with H&E. **e** Percentage of CD11c- and CD86- positive cells in TDLNs of naive and ICRP-KCC mice. **f**, **g** Proportion of CD3-, CD4- and CD8-positive cells in TDLNs (**f**) and peripheral blood (**g**) of naive and ICRP-KCC mice. **h** Histology from tumour rechallenge sites of naive and ICRP-KCC mice stained with H&E. Normal tissue (white arrows), tumour cells (red arrows), mitotic cells (black arrows), lymphocytes (blue arrows) and polymorphonuclear cells (green arrows). **i** Proportion of CD3-, CD4- and CD8-positive cells in tumour rechallenge sites of naive and ICRP-KCC mice. **j** Percentage of calcein-negative 4T1 cells left alone (Control), or co-cultured with splenocytes from naive or ICRP-KCC mice for 24 h (co-culture ratio 1:40). **k** Quantification of IFN-γ, TNF-α and IL-2 concentration in supernatants of co-cultures obtained as in (**j**), expressed as the means (±SD) of three independent experiments (*n* = 6 mice per group).

To investigate the effect of ICRP-KCC on the memory response in vivo, tumour-free mice that survived 150 days from a previous prophylactic vaccination with ICRP-KCC were rechallenged with viable 4T1 cancer cells, naive mice challenged with viable 4T1 cancer cells were used as control (Fig. 5a). The results show that tumour growth was prevented in rechallenged mice, while continuous tumour growth was observed in naive mice, challenged for the first time (Fig. 5b). This reflected in 100% (9/9) of survival in ICRP-KCC rechallenged mice, while naive mice perished by day 15 (9/9) (Fig. 5c). These results strongly suggest the stimulation of long-term antitumour immune memory by ICRP-KCC prophylactic vaccination.

To better characterise this immune response, we assessed tumour establishment, DC maturation, T-cell distribution and splenocyte–tumour-specific cytotoxicity after three days of tumour challenge (naive mice)/rechallenge (ICRP-KCC) (Supplementary Fig. 6). Histopathological analyses of tumour-draining lymph nodes showed a diffuse lymphoid hyperplasia in naive mice, whereas ICRP-KCC mice present a follicular lymphoid hyperplasia, indicating a modulated response associated with immunological memory (Fig. 5d). Due to these differences, we next evaluated the proportion of mature dendritic cells in tumour-draining lymph nodes. The results indicated that rechallenged ICRP-KCC group did not show significant difference in the percentage of DCs, but a higher proportion of mature DCs (CD11c+CD86+) were present when compared with challenged naive mice (Fig. 5e); on the other hand, studies of T-cell proportion in TDLNs revealed no differences in total CD3+ cells, a significant decrease of CD4+ T cells and an increase of CD8+ T cells in ICRP-KCC mice, when compared with naive mice (Fig. 5f).

Then, we assessed the proportion of T cells in peripheral blood, and no differences were detected in total CD3+ cells, while a significant decrease of CD4+ T cells and an increase of CD8+ T cells was observed in ICRP-KCC mice in contrast with naive mice (Fig. 5g).

Moreover, histopathological analyses of the tumour rechallenge site reveal that naive mice presented tumour establishment with an extensive infiltration of neoplastic cells in the striated muscle tissue and these cells demonstrated an intense mitotic activity (Fig. 5h left). On the other hand, ICRP-KCC mice showed a discrete infiltration of neoplastic cells in the striated muscle tissue, observing a strong infiltration of lymphocytes and polymorphonuclear cells (Fig. 5h right). Additionally, we observed an increase in total CD3+ cells, a significant decrease in CD4+ T cells and a significant increase in CD8+ T cells in the tumour-rechallenged site of ICRP-KCC mice, when compared with naive mice (Fig. 5i). Furthermore, splenocytes from ICRP-KCC mice mediated a cytotoxic effect in 4T1 cells, inducing loss of cell viability in up to 50% of cancer cells, whereas no significant cytotoxic effect was detected in 4T1 cells co-cultured with splenocytes from naive mice (Fig. 5j and Supplementary. Fig. 7), indicating a tumour-specific cytotoxicity. Finally, a significant increase in IFN-γ (95.05 ± 40.01 pg/mL), TNF-α (4239.85 ± 784.34 pg/mL) and IL-2 (20.97 ± 0.68 pg/mL) release was observed in the co-culture of 4T1 cells with splenocytes from ICRP-KCC mice, in comparison with the co-culture of 4T1 cells with splenocytes from naive mice (IFN-γ: 27.66 ± 15.12 pg/mL, TNF-α: 3310.46 ± 422.46 pg/mL and IL-2: 10.69 ± 2.94 pg/mL) (Fig. 5k), and no significant differences were detected in TH-2 cytokines, IL-4 and IL-5 release (Supplementary Table 2).

## Discussion

IMMUNEPOTENT CRP is a promising immunotherapy that induces regulated cell death in cancer cells. In T-acute lymphoblastic leukaemia (T-ALLL) cells (Molt-4 and CEM), ICRP provokes ROS-dependent apoptosis accompanied by mitochondrial and nuclear alterations.^11^ On the other hand, in cell lines from cervical (HeLa and SiHa cells) and lung cancer (A529 cells), ICRP induces non- apoptotic cell death (caspase-independent) that relies on ROS production, involving cell cycle arrest and loss of mitochondrial membrane potential.^9,10,13^ In this work, we demonstrated that ICRP caused caspase- and necrosome-independent regulated cell death that relies on ROS production in MCF-7, MDA-MB-231 and 4T1 cells, suggesting a conserved mechanism of action in solid tumours, and with some shared characteristics in all the cell lines tested to this day. Other treatments also induce different cell death mechanisms, being specific for the type of cancer cell line tested with shared characteristics in the cytotoxic pathway.^19,20^ In addition, no differences in cell death were observed in NEC-1 co-treated cells, which indicates that ICRP-mediated regulated cell death is different from necroptosis.

Moreover, inhibition of autophagy significantly potentiated ICRP-induced cell death, suggesting that ICRP induces pro-survival autophagosome formation in MDA-MB-231 and 4T1 cells, corresponding with our previous reports in MCF-7.^13^ The induction of autophagy as a protective mechanism in cancer cells has also been demonstrated with other cancer treatments, including chemotherapy and radiation.^21^ Furthermore, inhibition of caspases also resulted in a significant augmentation of ICRP-mediated cell death in MDA-MB-231 cells and 4T1 cells, but not MCF-7 cells. These results could be related with the “Phoenix Rising” pathway of cell death-induced tumour repopulation, in which caspase 3 plays key roles that have been demonstrated in 4T1 and MDA-MB-231 cells, and confirmed using MCF-7 cells, which are deficient in caspase 3 expression.^22^

Additionally, ICRP induced P-eIF2α (ER stress biomarker) in the three cell lines. The capacity of many agents to induce ICD relies on their ability to trigger ROS production and ER stress, being these two cellular processes essential components that initiate the intracellular danger signalling pathways that dictate ICD.^23,24^

ER stress response and ROS production have been associated with the induction of autophagy.^24,25^ As we mentioned before, in this study, we proved that ICRP caused autophagosome formation in MCF-7, MDA-MB-231 and 4T1 cells, corresponding with the decrease in p62 levels observed after ICRP treatment, as p62 is itself degraded, when autophagy is induced.^26^ The induction of ROS production, ER stress and autophagy has been observed in other studies, for instance, Shikonin induces cell death and pro-survival autophagy in human melanoma cells via ROS-mediated ER stress.^27^ Furthermore, radiotherapy causes ROS generation, DNA damage, ER stress and autophagy in cancer cells.^28^ The increase in ROS, ER stress and autophagy has been associated with cancer therapies that are classified as ICD inducers.^29,30^

P-eIF2α regularly antecedes chaperone protein exposure in the course of ICD; nonetheless, P-eIF2α is not necessarily followed by CRT exposure, particularly when the ER stress response re-establishes cell homoeostasis;^24,31^ for this reason, we decided to evaluate P-eIF2α after 18 h of treatment, and surface-exposed chaperones after 24 h of treatment with ICRP. Here, we demonstrated that ICRP induced P-eIF2α and increased the “eat me signals” CRT, HSP70 and HSP90 on breast cancer cell surface, and also decreased the surface-associated “don´t eat me” signal CD47. This constellation of surface signals then acts on phagocytic receptors of immune cells facilitating cellular engulfment.^32^ The increased exposure of CRT, HSP70 and HSP90 proteins on cell surface was also observed after EPI treatment, a well-known inducer of ICD in breast cancer cells,^33^ used here as positive control.

Higher levels of ATP release were observed after ICRP and EPI treatment in MCF-7 and MDA-MB-231 cells in comparison with 4T1 cells, this corresponds with previous studies in which it has been demonstrated that 4T1 is a p53-deficient cell line^34^ and ATP is produced at higher levels using oxidative phosphorylation in cells expressing p53 versus cells lacking p53.^35^ Besides, ICRP and EPI treatments triggered significant HMGB1 release in MCF-7 and MDA-MB-231, but partial HMGB1 release in 4T1 cells. This low ICRP-mediated HMGB1 release could be due to the exposure of cells to ICRP CC_50_ treatment, which was not sufficient for a significant release of HMGB1, as some agents induce the release of HMGB1 at CC_100_ but not CC_50_.^36^ Furthermore, as HMGB1 release has been observed at late times in the course of ICD,^37^ we evaluated this parameter at 48 h, and we found a significant HMGB1 release after ICRP and EPI treatment on the three cell lines.

Another drug classified as ICD inducer is the alkylating agent CPA;^38,39^ controversially, in our hands, this drug only triggered DAMP emission in MCF-7 cells but not in triple-negative MDA-MB-231 and 4T1 cells, this could be due to the fact that alkylating agents can induce NRF2 activation that blocks ER stress,^40^ one of the principal parameters related to ICD.

DAMP emission induced by ICRP was also observed in MCF-7 cells, which are deficient in caspase 3 expression, suggesting that these processes do not require caspase 3 activation to take place. Although caspase activation plays a key role during DAMP emission and the immunogenicity of the cell death induced by several cancer treatments,^23,41,42^ it has also been observed that a DAMP emission can be triggered in a caspase-independent fashion, for instance, Hyp-PDT-induced CRT exposure is caspase-independent,^43^ and chemotherapeutic agents can activate an alternative caspase-insensitive mechanism for secretion of ATP.^44^

Nevertheless, it has been demonstrated that the release/exposure of all DAMPs is not determinant for immunogenic cell death;^45^ thus, we further evaluated ex vivo and in vivo the immunogenicity of the cell death induced by ICRP. Cancer-dying cells by ICD inducers promote DC maturation, which strongly activates anticancer immunity. According to current models, only few treatments induce intracellular signalling pathways that lead to a cancer cell death able to stimulate fully mature DCs, including cyclophosphamide,^46^ γ-irradiation,^47^ doxorubicin, oxaliplatin,^48^ bortezomib^49^ and a CD47 agonist peptide.^50^ Other therapies are only speculated to induce complete DC maturation, or use LPS, IFN type 1 or other stimulants in combination with tumour cell lysates (TCL) to promote DC maturation.^29,46^ In addition, some cancer treatments may cause semi-mature DCs, namely DCs that lack phenotypic maturation markers or cytokine release, and thereby are unable to efficiently prime T cells.^51^ Here, we demonstrated that ICRP-KCC obtained from ICRP-mediated 4T1 cell death induced maturation of BMDCs, which triggers a specific anticancer immune response against 4T1 cancer cells.

Furthermore, with the prophylactic tumour vaccination model, we demonstrated that ICRP-KCC activates the adaptive immune system in 90% BALB/c mice, leading to a long-term antitumour memory, which is desirable in cancer patients dealing with cancer recurrence. Usually, ICD inducers protect from 50% to 90% of individuals when used alone, without any type of adjuvants; such is the case of Hypericin-based photodynamic therapy (87%),^43^ mitoxantrone (80%),^52^ oxaliplatin (80%)^53^ and nanosecond pulsed electric fields (50%).^54^ Other treatments need two previous vaccinations to induce slower tumour growth in vaccinated mice^55^ or the use of combinational therapy to reach protection in 80% of the cases.^56^ Moreover, agents classified as ICD inducers have been studied using TCL-loaded dendritic cell vaccines, but not TCL, and after several vaccine boosting, they reach up to 70% of survival.^29^

To test the potential of ICRP to trigger ICD on breast cancer cells in contrast to other well-known ICD inducers, we compared ICRP treatment with EPI and CPA (two drugs classified as ICD inducers^33,38,39^) in prophylactic vaccination assays. We found that whereas EPI-KCC vaccination protected 70% of individuals, CPA-KCC vaccination only protected 20% of individuals. These results are consistent with the observation of higher DAMP emission triggered by ICRP as compared to EPI, and the absence of DAMP emission after CPA treatment in 4T1 cells. Our results highlight the importance of evaluating the immunogenicity of the cell death provoked by ICD inducers in different biochemical contexts, as the immune effect of ICD drugs, such as CPA, can differ between cancer models.

It is known that T-cell responses generally peak ∼1–4 days after a second antigen stimulation.^57,58^ Here, we analysed tumour-draining lymph nodes after three days of tumour rechallenge and observed a follicular lymphoid hyperplasia in ICRP-KCC mice, which is associated with immunological memory; additionally, we observed an increase of mature DCs in ICRP-KCC mice in comparison to the naive group. Several studies have demonstrated that effector memory T cells potentiate the maturation of DCs, and in addition to T cells, BCR signalling is sufficient for memory B cells to induce complete activation of DCs.^59^

We also observed an increase of CD8+ T cells over CD4+ T cells in the TDLNs, peripheral blood and tumour rechallenge site of immunised mice; these results correspond with the observations that in the same host, memory assessments result in robust CD8+ T-cell responses, but poor boosting of CD4+ T-cell recall responses,^60^ which is correlated with the demonstration that CD4+ memory cells proliferated for a shorter period of time than CD4+ naive cells because of their cytokine profile.^61^

Finally, we observed a tumour-specific cytotoxicity by splenocytes from immunised mice, whereas no cytotoxicity was observed in the co-culture of 4T1 with splenocytes from naive mice, indicating the activation of a rapid immune response triggered by the antitumour memory establishment. This evaluation corresponds with our ex vivo assessment where we observed an increase in Th1-type cytokines, which are associated with the generation of cytotoxic responses.^62^

The results in this work are supported by the recent demonstration that breast tumour-bearing mice treated with ICRP present a decrease in tumour volume and increase in survival in comparison with untreated mice.^63^ Also, within the tumour, ICRP treatment decreased PD-L1, IDO and Gal-3 expression, IL-6, IL-10 and MCP-1 levels, and increased IFN-γ and IL-12 levels. Moreover, ICRP treatment increased CD8+ T cells, memory T cells and innate effector cells in peripheral blood, where an increase was also observed in IFN-γ and IL-12 levels,^63^ indicating that these findings could be due to ICD induction in the tumour of these mice.

**Fig. 6:**
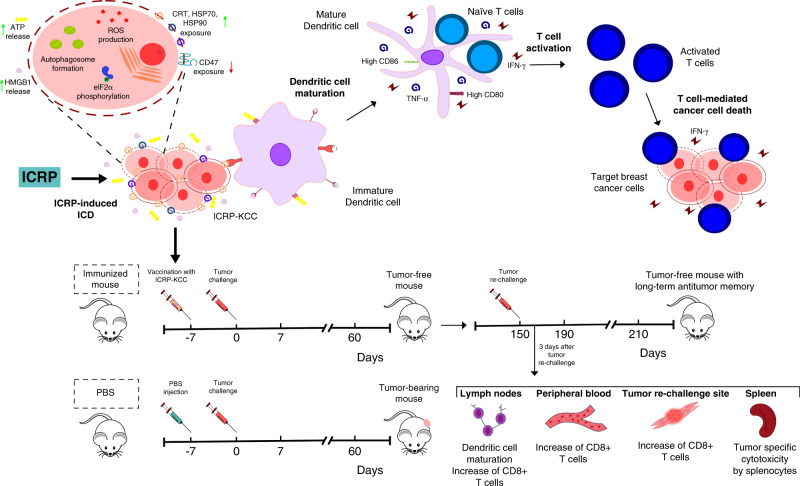
Schematic depiction of IMMUNEPOTENT CRP-induced immunogenic cell death in breast cancer cells. IMMUNEPOTENT CRP induces ROS production, autophagosome formation and eIF2α phosphorylation in breast cancer cell lines leading to DAMP release. Neoantigens and the release of DAMPs promote DC maturation, which triggers T-cell activation to induce cancer cytotoxicity. Moreover, ICRP-KCC prophylactic vaccination prevented tumour establishment and induces long-term antitumour memory in BALB/c mice; this protection involves an increase of DC maturation in lymph nodes, CD8+ T cells in lymph nodes, peripheral blood and tumour rechallenge site, as well as tumour-specific cytotoxicity by splenocytes. ROS, reactive oxygen species; ER, endoplasmic reticulum; CRT, calreticulin; HSP70, 70-kDa heat shock protein; HSP90, 90-kDa heat shock protein; CD47, Cluster of Differentiation 47; HMGB1, high-mobility group box 1; ATP, adenosine triphosphate; TNF-α, tumour necrosis factor alpha; IFN-γ, interferon gamma.

In conclusion, IMMUNEPOTENT CRP triggers loss of mitochondrial membrane potential, pro-survival autophagosome formation, eIF2α phosphorylation and caspase-independent but ROS-dependent cell death in breast cancer cells; these intracellular signalling pathways lead to alterations in the composition of the plasma membrane of dying cells (increased CRT, HSP70 and HSP90 exposure and decreased CD47 exposure), as well in their microenvironment (release of ATP and HMGB1), which stimulates DC maturation, priming of T cells, promoting an antitumour immune response ex vivo and in vivo and leading to a long-term antitumour memory (Fig. 6). Overall, our results show that ICRP may have the capacity to turn breast cancer cells into potential vaccines in vivo.

## Supplementary information

Supplementary Data

## Data Availability

The datasets that support the findings of this study are available from the corresponding author on reasonable request.
