# Supplementary material for: The bovine dialysable leukocyte extract IMMUNEPOTENT CRP induces immunogenic cell death in breast cancer cells leading to long-term antitumour memory
Source: Br J Cancer. 2021 Feb 3;124(8):1398–410. doi: 10.1038/s41416-020-01256-y (PMC8039030; doi:10.1038/s41416-020-01256-y)
Supplement: Supplementary file 1 — Supplementary Data [file 41416_2020_1256_MOESM1_ESM.pdf]

SUPPLEMENTARY DATA

The bovine dialyzable leukocyte extract IMMUNEPOTENT CRP induces immunogenic cell death in breast cancer cells leading to long-term antitumor memory.

Running title: IMMUNEPOTENT CRP induces immunogenic cell death.

Alejandra Reyes-Ruiz[1]*, Kenny Misael Calvillo-Rodriguez[1]*, Ana Carolina Martínez-Torres[1], Cristina Rodríguez-Padilla[1,2]

[1]Universidad Autónoma de Nuevo León, Facultad de Ciencias Biológicas, Laboratorio de Inmunología y Virología, México.

[2]Longeveden, SA de CV, México.

Corresponding Author: Ana Carolina Martínez-Torres.

ORCID: 0000-0002-6183-0089

Universidad Autónoma de Nuevo León, Facultad de Ciencias Biológicas, Laboratorio de Inmunología y Virología, México. ana.martinezto@uanl.edu.mx

*These authors contributed equally to this work.

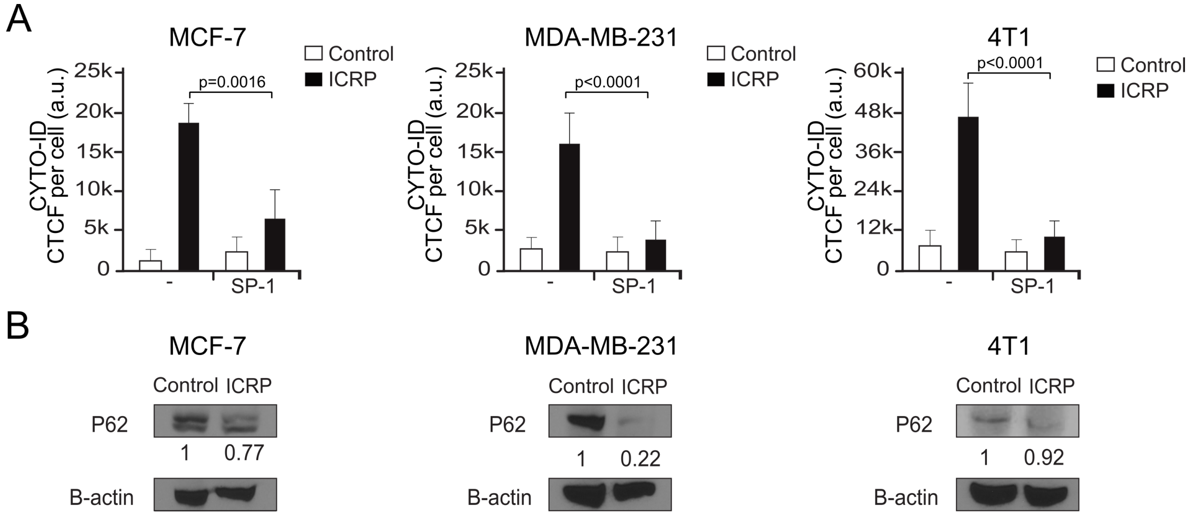

Supplementary Figure 1. A. Corrected total cell fluorescence (CTCF) of CYTO-ID staining shown in arbitrary units (a.u.) in MCF-7, MDA-MB-231, and 4T1 cells left untreated (control) or treated with ICRP CC_{50} for 24 h without co-treatment (-) or co-treated with Spautin-1 (SP-1). The means (± SD) of triplicates of at least three independent experiments were graphed. **B.** Western blot analysis of cell lysates prepared from MCF-7, MDA-MB-231, and 4T1 cells left untreated (control) or treated with ICRP CC_{50} for 24 h showing p62 expression. Relative densitometric values are provided below the blot images. The same blot was reproved with β-actin to confirm equal loading of each lane.

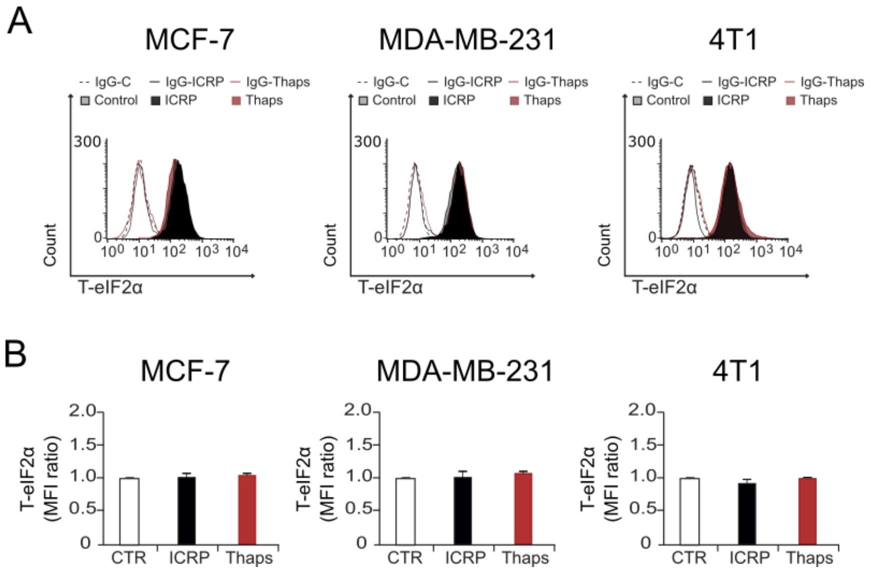

Supplementary Figure 2. A. Representative FACS histograms of T-eIF2α staining (filled histograms) and IgG isotype antibodies (open histograms) of cancer cells left untreated (negative control in gray) or treated with ICRP CC_{50} for 18 h (in black) or 1 µM Thaps for 2h (positive control in red). **B.** Charts are the quantification of T-eIF2α staining in cancer cells left untreated or treated with ICRP CC_{50} for 18 h or 1 µM Thaps for 2h. Graphs represent the means (± SD) of triplicates of at least three independent experiments.

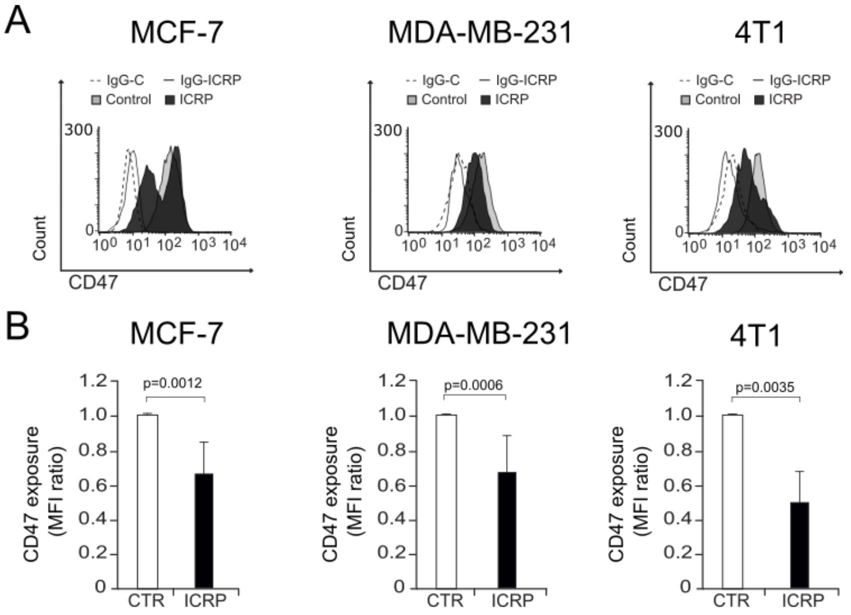

Supplementary Figure 3. A. Representative FACS histograms of CD47 exposure (filled histograms) and IgG isotype antibodies (open histograms) of cancer cells left untreated (negative control in gray) or treated with ICRP CC_{50} (in black) for 24 h. **B.** Charts are the quantification of CD47 exposure in cancer cells left untreated or treated with ICRP CC_{50} for 24 h. Graphs represent the means (± SD) of triplicates of at least three independent experiments.

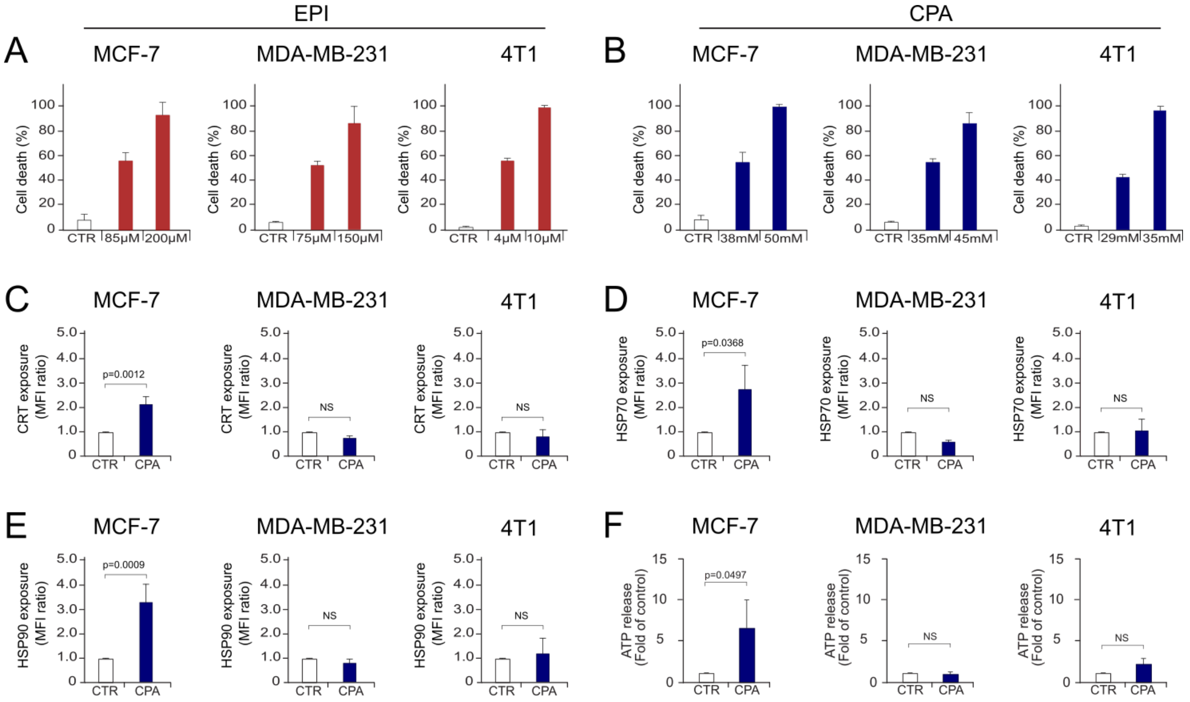

Supplementary Figure 4. A-B. Quantifications of cell death in MCF-7, MDA-MB-231 and 4T1 cells treated with different concentrations of EPI (A) or CPA (B) for 24 h. **C-E.** Quantification of CRT (C), HSP70 (D) or HSP90 (E) exposure in cancer cells left untreated or treated with CPA CC_{50} for 24 h. **F.** Quantification of ATP release through bioluminescence detection in the supernatants of cancer cells in absence (negative control) or presence of CPA CC_{50} for 24 h. Graphs represent the means (± SD) of triplicates of at least three independent experiments.

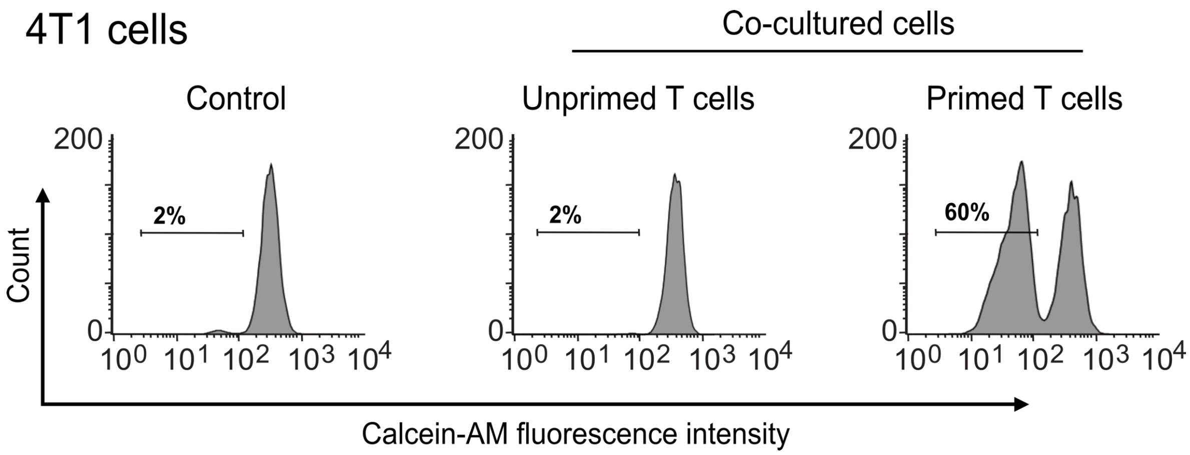

Supplementary Figure 5. Representative flow cytometry histograms showing the percentage of calcein negative 4T1 cells left alone (Control), or in co-culture ratio 1:5 with unprimed T-lymphocytes (previously co-cultured with unstimulated BMDCs) or primed T-lymphocytes (previously co-cultured with BMDCs-ICRP-KCC) for 24 h.

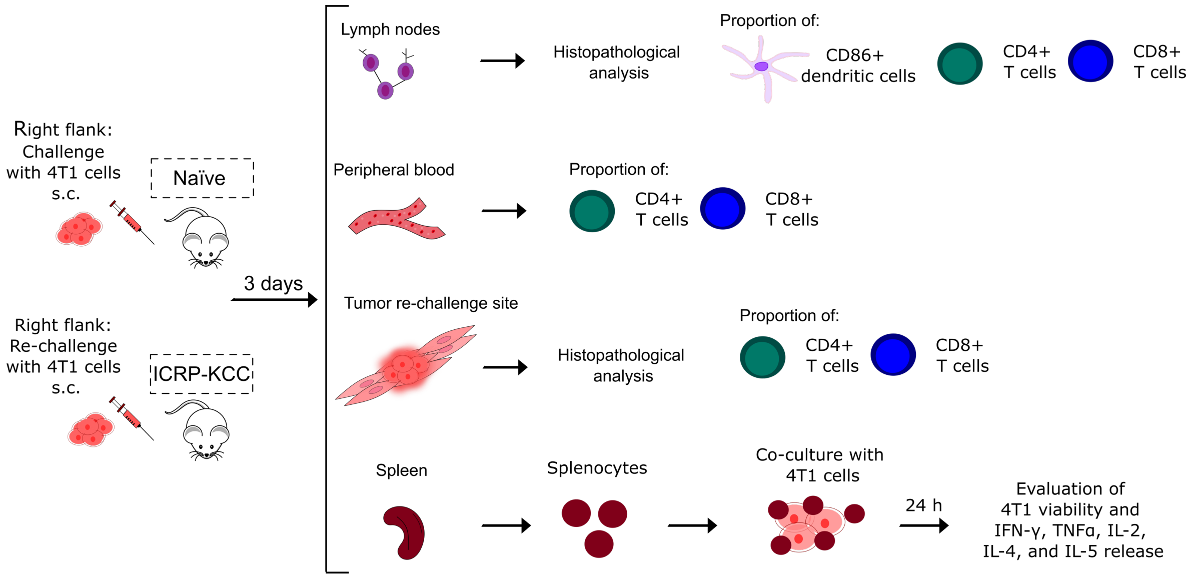

Supplementary Figure 6. Naïve mice (n=6) and mice in remission after ICRP-KCC prophylactic vaccination (n=6) were challenged / re-challenged s.c. with $5x10^5$ viable 4T1 cells. Three days later, tumor-draining lymph nodes, peripheral blood, tumor re-challenge site, and spleen were obtained to perform histopathological analyses on TDLNs and tumor re-challenge site; proportion of CD86+ dendritic cells in TDLNs; proportion of T cells in TDLNs, peripheral blood and tumor re-challenge site; and evaluation of tumor specific cytotoxicity by splenocytes**.**

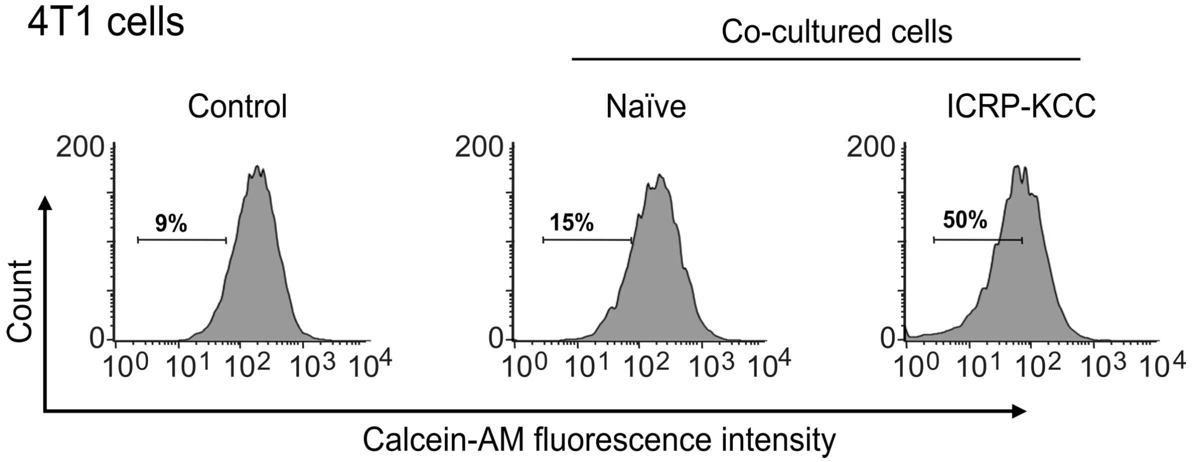

Supplementary Figure 7. Representative flow cytometry histograms showing the percentage of calcein negative 4T1 cells left alone (Control), or after 24 h of co-culture with splenocytes of naïve mice or mice in remission after ICRP-KCC prophylactic vaccination after 3 days of tumor re-challenge. Co-culture ratio 1:40**.**

Supplementary Table 1. IL-4 and IL-5 release in co-cultures of T cells with Control- or ICRP-KCC-pulsed BMDCs and co-cultures of 4T1 cells with Unprimed- or BMDCs-ICRP-KCC primed-T cells.

Cytokine	BMDCs-Control + Unprimed-T cells	BMDCs-ICRP-KCC + Primed-T cells
IL-4	0 ± 0	0.31 ± 0.54
IL-5	2.3 ± 4.09	1.01 ± 1.74
Cytokine	4T1 cells + Unprimed-T cells	4T1 cells + Primed-T cells
IL-4	0.75 ± 1.31	0.60 ± 1.05
IL-5	0.15 ± 0.27	0 ± 0

Supplementary Table 2. IL-4 and IL-5 release in co-cultures of 4T1 cells with splenocytes from naïve or ICRP-KCC mice.

Cytokine	4T1 cells + splenocytes naive	4T1 cells + splenocytes ICRP-KCC
IL-4	4.99 ± 0.22	5.52 ± 0.70
IL-5	0 ± 0	1.74 ± 4.18
